# Relay Positions Considering Interference from Other Sub-Channels in OFDMA-Based D2D Group-Casting Systems †

**DOI:** 10.3390/s19061374

**Published:** 2019-03-19

**Authors:** Minjoong Rim, Eulhyeon Go

**Affiliations:** Department of Information and Communication Engineering, Dongguk University, Seoul 04602, Korea, dmfgus5118@naver.com

**Keywords:** D2D, Group-casting, OFDMA, Relay, In-band emission, Interference

## Abstract

Device-to-device (D2D) communication is a technique for direct communication between devices without going through a base station or other infrastructure. D2D communication technology has the advantages of improving spectrum efficiency and reducing transmission delay and transmission power. In D2D communication systems, orthogonal frequency-division multiple access (OFDMA) is widely used to maintain similarities with cellular communication systems and to secure transmission distance. OFDMA allows flexible and efficient use of frequency resources by allocating sub-channels independent to each user. In this paper, we consider a D2D overlay system that uses different sub-channels for cellular and D2D communications. In theory, the signals on different sub-channels of an OFDMA system are orthogonal and not interfered with each other. However, in a D2D communication system, which operates in a distributed manner, there is non-negligible interference from other sub-channels because of in-band emissions. In this paper, we address the performance degradation resulting from the interference from other sub-channels for OFDMA-based D2D group-casting systems. We consider three different scenarios of D2D relay, and we find the relay position that minimizes the outage probability. The simulation and analytical results show that the optimal location of a relay can be considerably different according to the source location and the target scenario.

## 1. Introduction

Recently, there is growing interest in device-to-device (D2D) communication technologies capable of directly transmitting data between devices without going through a base station or other infrastructure [1,2,3,4,5,6]. D2D communication technology has the advantage of being able to communicate even when there is no base station around or the base station is not operational for various reasons. It can also improve the efficiency of spectrum utilization and help to solve traffic overload problems of the base stations from the surge of mobile traffic. Furthermore, due to the proximity of devices, D2D communication can reduce the transmission power of devices, which is important for many applications including the Internet of things (IoT) or sensor devices [7,8,9]. D2D communication is considered one of the key technologies for 5G communication systems for enabling IoT, vehicle to everything (V2X), public safety, data offloading, and social-aware communications [10,11,12,13]. 

Orthogonal frequency-division multiple access (OFDMA) is one of the most widely used multiple access technologies in cellular communication systems [14]. OFDMA allows flexible and efficient use of frequency resources by allocating sub-channels, which is comprised of subcarriers, to each user. OFDMA is also widely used in D2D communication systems to maintain the similarities with cellular communication systems and to ensure transmission distance [15,16,17,18]. D2D underlay systems operate cellular and D2D communications on the same sub-channel of the OFDMA system [19,20,21]. Although the performance of D2D underlay systems can be improved by maximizing frequency reuse, complicated interference management may be required. Above all, if the interference channel measurement and the interference control are not perfect, D2D communication may not be guaranteed or the performance of the cellular system may be degraded. In this paper, we consider a D2D overlay system that uses different sub-channels for cellular and D2D communications. Theoretically, signals on different sub-channels in OFDMA systems are orthogonal and not interfered with each other. However, in an actual system, there is non-negligible interference from other sub-channels that results from in-band emissions [22,23,24,25]. Unlike a cellular communication system in which a base station (BS) is centered on a cell, a D2D communication system operates in a distributed manner, and the interference from other sub-channels can be considerable, especially when the number of sub-channels in OFDMA systems is large. 

This paper assumes a D2D overlay system to focus on the effects of other-channel interference and does not consider co-channel interference. However, if a D2D underlay system is considered in order to increase frequency reuse, control for co-channel interference is also required. Efficient interference management schemes for co-channel interference can be found in many papers [26,27,28,29,30] and can be used in addition to the techniques discussed in this paper.

This work deals with the interference from other sub-channels in OFDMA-based D2D systems. This journal article extends the conference paper [31] by considering several different scenarios of D2D configurations, while the preliminary version of this paper focused on a single limited scenario with limited assumptions. It also includes more details on the system model, assumptions and numerical analysis, as well as additional simulation results.

One of the most widely used communication configurations in D2D communication systems is group-casting [32,33,34]. In group-casting, one transmitter within a group transmits data to the remaining devices in the group. However, if the transmitter is at the edge of the group or near the edge, the direct transmission from the transmitter can cause a high outage probability, and a relay in the right position can be used to reduce the outage [35,36,37]. The relay receives the data from the transmitter and passes the data to other devices in the group to enable reliable data transmission. Using a relay can also extend the transmission range or reduce the transmit power, which results in low interference to cellular devices or other D2D devices [35,36,37]. In OFDMA-based D2D systems, transmission through a relay should consider the interference from other sub-channels, but most studies do not take care of the interference. 

In this paper, we consider relay positions in a D2D group to minimize the outage probability from the interference from other sub-channels while ignoring other performance-degradation effects such as collisions, fading, or co-channel interference. When a transmitter sends data to multiple receivers in a group, the outage probabilities may not be sufficiently small, since the signal-to-interference ratio (SIR) can be low for receivers far away from the transmitter or close to interferers in other sub-channels. In order to reduce the outage probabilities, we consider repetitive transmission through a relay in addition to the direct transmission between the transmitter and receivers. In this paper, we construct three different relay scenarios considering the interference from other-channel interferers in the D2D group-casting system. We calculate outage probabilities resulting from the interference from other sub-channels, and we find the relay position achieving the lowest outage probability. 

The rest of the paper is organized as follows. Section 2 presents the system models and three scenarios for D2D group-casting with a relay. Section 3 addresses the outage probabilities for the three scenarios considering the interference from other sub-channels. The numerical results with various transmitter and relay positions in a D2D group are given in Section 4, and finally, Section 5 draws the conclusions. 

## 2. System Model

### 2.1. Device-To-Device (D2D) Group-Casting

**Figure 1:**
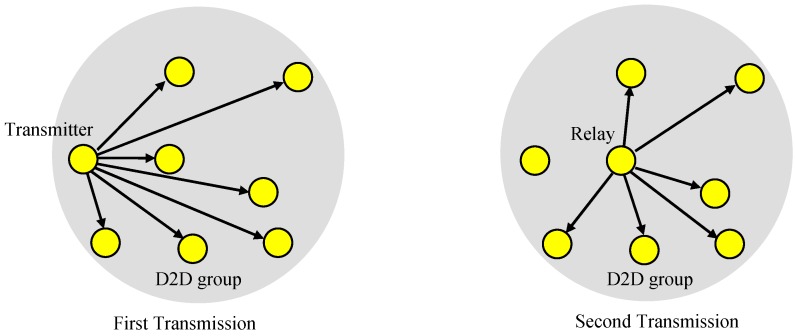
Device-to-device (D2D) group-casting with a relay.

In the paper, we considered a case where one transmitter in a D2D group transmits data to the remaining devices in the group, as shown in Figure 1. The signal transmitted from the transmitter is broadcast to all other devices in the group. However, especially when the transmitter is at the edge of the group or near the edge, the success probability of the receivers may not be large enough, and a relay can be used to improve the success rate. The relay sends the received data to the other devices in the group. 

**Figure 2:**
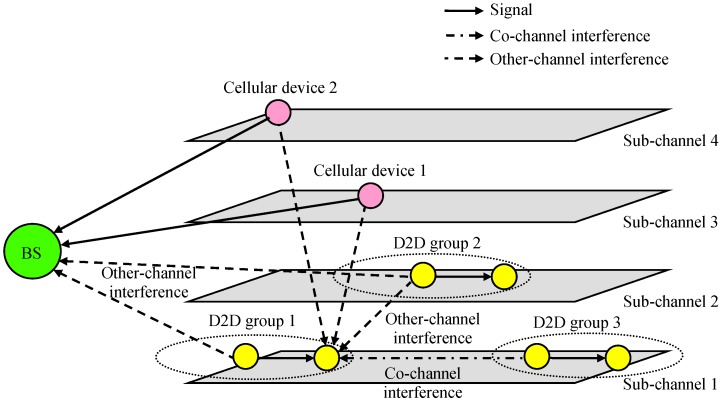
Other-channel interference.

We also considered a D2D overlay system that uses different sub-channels for cellular and D2D communications, as shown in Figure 2. Different D2D groups can be assigned to different sub-channels to reduce the interference between each other, or they can be allocated to the same sub-channel if they are geographically separated or some contention resolution mechanism such as listen-before-talk (LBT) is employed. In this paper, we assumed that co-channel interference occurring in the same sub-channel can be resolved or ignored, and we focused on other-channel interference from other sub-channels. 

In general, D2D communication uses the uplink, and other-channel interference from D2D transmitters to the BS can be resolved by limiting the transmit power of D2D transmitters. If we reduce the transmit power of D2D transmitters, however, the effect of the other-channel interference from the cellular system to D2D receivers can become more severe. In this paper, we analyzed other-channel interference from cellular devices to D2D receivers. 

This paper considered a D2D group-casting system with a focus on interference from other sub-channels. In this paper, we ignored other performance-degradation effects such as collisions between intra-group devices, insufficient signal power due to fading, or co-channel interference from other D2D groups or cellular devices on the same sub-channel. We assumed that received signal power was sufficiently larger than the noise power, and fading effects can be ignored because there was sufficient diversity in the channels. We also assumed that smart resource management techniques can be used to solve the collisions in the same D2D group and to alleviate the co-channel interference from other D2D groups or cellular devices in the same sub-channel. Nevertheless, it may be difficult to avoid the interference from other sub-channels, unless very complicated resource management schemes over multiple sub-channels are used. 

In this paper, we only considered interference from D2D or cellular devices on other sub-channels for the cause of reception failures, assuming that there is one D2D or cellular transmitter in each sub-channel in the target cell area. The interference from other sub-channels is very weak compared to co-channel interference, thus, only receivers near the other-channel interferers are affected. However, the interference from other sub-channels cannot be ignored, especially when the transmission power of devices in other sub-channels is high or the number of sub-channels is large. In order to alleviate performance degradation from the interference, a relay can be used, and we considered three different scenarios using a relay.

### 2.2. Scenario 1: Single Transmission through a Relay

**Figure 3:**
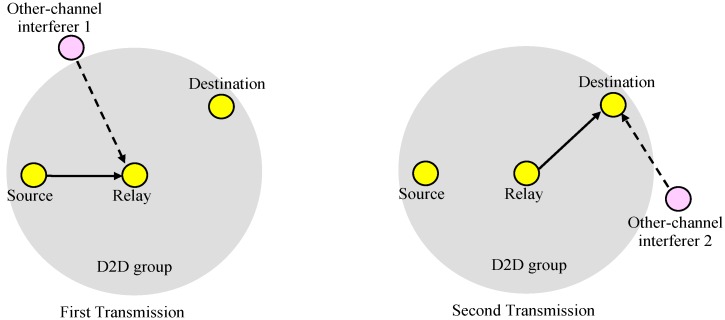
Scenario 1.

In the first scenario, as shown in Figure 3, the source (transmitter) passes data to the relay and the relay transmits to the destinations (receivers) in the group if the relay receives the data successfully. In the first scenario, we did not consider the direct link from the source to the destinations, and the destinations received data only for the second transmission phase. The reception at a destination is successful if both the source-to-relay and relay-to-destination transmissions are successful. We assumed that in the cell area there was one interferer per sub-channel, which may be a D2D or cellular transmitter. We also assumed that the locations of interferers were independent on the sub-channels or transmissions. 

### 2.3. Scenario 2: Repeated Transmission through a Relay

**Figure 4:**
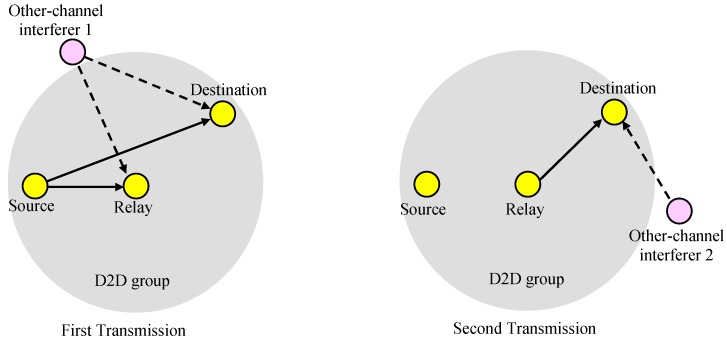
Scenario 2.

In the second scenario, as shown in Figure 4, the source transmits data to the destinations in the group, and the relay repeatedly sends the received data to the destinations if the transmission from the source to the relay is successful. If the relay fails to receive, there is no second transmission. The destination receives the data successfully if the source-to-destination transmission is successful, or both the source-to-relay and relay-to-destination transmissions are successful. 

### 2.4. Scenario 3: Repeated Transmission through a Successful Relay

**Figure 5:**
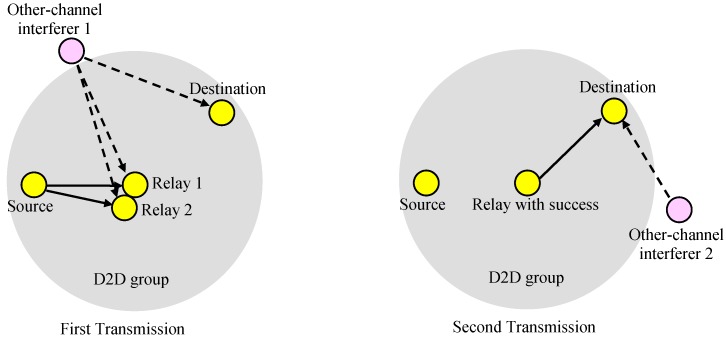
Scenario 3.

The third scenario, as shown in Figure 5, is similar to the second scenario, but we assumed that there were multiple candidates for a relay, and the candidates that successfully received the data acted as a relay. Since there can be multiple candidates that receive the data successfully, some resolution mechanism is required to make only one act as a relay. 

**Figure 6:**
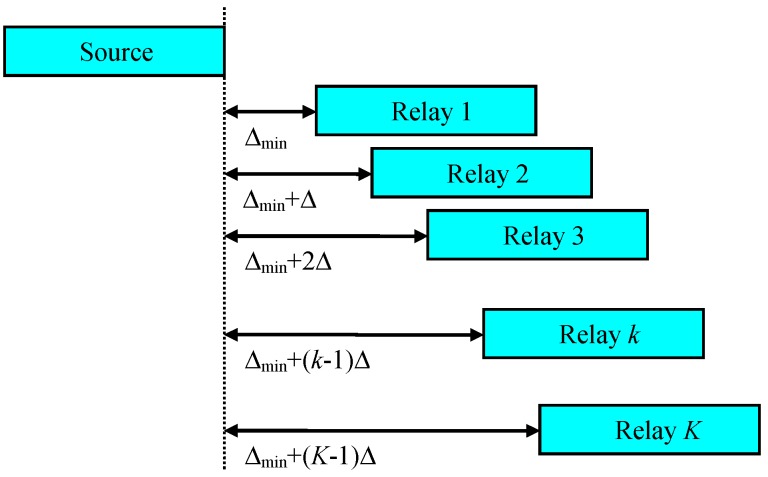
Listen-before-talk (LBT) with different delays.

For example, one can use LBT and different delays before starting transmission in order to select only one successful relay, as shown in Figure 6. Suppose that there are K candidate relays. If a candidate relay fails to receive the packet from the source, it does not transmit. Upon successfully receiving the packet from the source, the $k^{th}$ (k=1,2,⋯,K) candidate relay waits $\Delta_{\min}+(k-1)\Delta$ seconds and performs carrier sensing. It can start transmission if no other candidate relay is transmitting, meaning that candidate relays between 1 and k−1 will fail to receive. Based on this kind of mechanism, one candidate relay (at most) can send the packet. 

There is some chance that any of the candidate relays may not succeed. If the probability that a candidate relay fails to receive from the source is $P_{SR}^{Outage}$, then the probability that all K candidate relays fail is ${\left(P_{SR}^{Outage}\right)}^{K}$. This value might be ignored when calculating the final outage probability, especially when K is large and $P_{SR}^{Outage}$ is small. 

For simplicity, we assumed that one (and only one) relay can transmit for this scenario. The reception at a destination is successful if the source-to-destination transmission is successful or the relay-to-destination transmission is successful. 

## 3. Outage Probabilities

### 3.1. Outage Probabilities

Consider an OFDMA-based D2D system in a circular cell with radius R. Suppose that there are M sub-channels, and one (and only one) D2D or cellular device transmits for each sub-channel at a time. When considering the transmission on the $n^{th}$ sub-channel, the other M−1 sub-channels produce other-channel interference to the $n^{th}$ sub-channel. Let the interference effect resulting from in-band emission from the $m^{th}$ sub-channel to the $n^{th}$ sub-channel be $\lambda_{m,n}$, where $\lambda_{m,n}=1$ if m=n and $0<\lambda_{m,n}<<1$ otherwise. The amount of interference from other sub-channels is determined by $\lambda_{m,n}$ (m≠n). 

**Figure 7:**
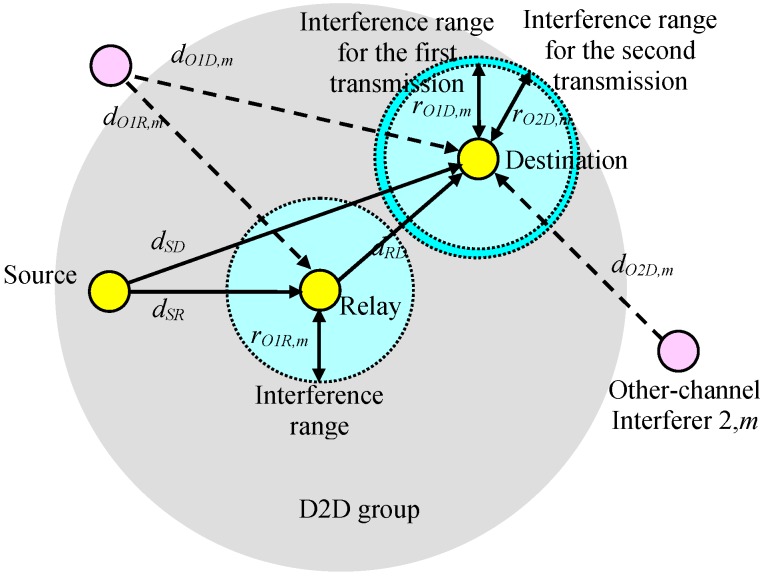
Interference from other sub-channels.

We considered relay-based D2D transmission consisting of a source (transmitter), a destination (receiver), and a relay on the $n^{th}$ sub-channel, as shown in Figure 7. In the first transmission phase, the source transmits data and in the second transmission phase, the relay passes the received data to the destination. Let the other-channel interferer 1,m and 2,m be the interferers on the $m^{th}$ sub-channel (m≠n) for the first (source-to-relay) and the second (relay-to-destination) transmission phases, respectively. 

Let the positions of the source, relay, destination, other-channel interferer 1,m, and other-channel interferer 2,m be $\left(x_{S},y_{S}\right)$, $\left(x_{R},y_{R}\right)$, $\left(x_{D},y_{D}\right)$, $\left(x_{O1,m},y_{O1,m}\right)$, and $\left(x_{O2,m},y_{O2,m}\right)$, respectively. $d_{SD}=\sqrt{{\left(x_{S}-x_{D}\right)}^{2}+{\left(y_{S}-y_{D}\right)}^{2}}$ represents the distance between the source and the destination, $d_{SR}=\sqrt{{\left(x_{S}-x_{R}\right)}^{2}+{\left(y_{S}-y_{R}\right)}^{2}}$ represents the distance between the source and the relay, $d_{RD}=\sqrt{{\left(x_{R}-x_{D}\right)}^{2}+{\left(y_{R}-y_{D}\right)}^{2}}$ is the distance between the relay and the destination, $d_{O1R,m}=\sqrt{{\left(x_{O1,m}-x_{R}\right)}^{2}+{\left(y_{O1,m}-y_{R}\right)}^{2}}$ represents the distance between other-channel interferer 1,m and the relay, $d_{O1D,m}=\sqrt{{\left(x_{O1,m}-x_{D}\right)}^{2}+{\left(y_{O1,m}-y_{D}\right)}^{2}}$ is the distance between the other-channel interferer 1,m and the destination, and $d_{O2D,m}=\sqrt{{\left(x_{O2,m}-x_{D}\right)}^{2}+{\left(y_{O2,m}-y_{D}\right)}^{2}}$ is the distance between the other-channel interferer 2,m and the destination. $P_{S}^{TX}$ is the transmission power of the source, $P_{R}^{TX}$ is the transmission power of the relay, $P_{O1,m}^{TX}$ is the transmission power of the other-channel interferer 1,m, and $P_{O2,m}^{TX}$ is the transmission power of the other-channel interferer 2,m. 

Consider the transmission from the source to the destination. The received signal power at the destination, dented as $P_{SD}^{RX}$, can be represented as:

(1) $$P_{SD}^{RX}=K_{1}P_{S}^{TX}{d_{SD}}^{-\alpha },$$

where $K_{1}$ is a constant and α is the path-loss exponent parameter between two devices. The received interference power from the other-channel interferer 1,m at the destination is denoted as:

(2) $$P_{O1D,m}^{RX}=K_{1}\lambda_{m,n}P_{O1,m}^{TX}{d_{O1D,m}}^{-\alpha },$$

and the SIR at the destination considering the $m^{th}$ channel interferer only, denoted as $SIR_{SD,m}$, can be written as follows:

(3) $$SIR_{SD,m}=\frac{P_{SD}^{RX}}{P_{O1D,m}^{RX}}=\frac{P_{S}^{TX}{d_{SD}}^{-\alpha }}{\lambda_{m,n}P_{O1,m}^{TX}{d_{O1D,m}}^{-\alpha }}.$$

Assuming that α is large and, thus, the nearest interferer dominates the amount of interference, we can only consider the interference from the nearest interferer, or more precisely, we can consider the $m^{th}$ channel interferer, where $m=\underset{m^{\prime }}{\arg\max}\left\{\lambda_{m^{\prime },n}{d_{O1D,m^{\prime }}}^{-\alpha }\right\}$. The interference range at the destination resulting from the interference from the $m^{th}$ sub-channel, expressed as *r*_*O*1*D*,*m*_, can be defined as the minimum $d_{O1D,m}$ that satisfies *SIR*_*SD*,*m*_ ≥ Γ_D_, where $\Gamma_{D}$ is the required SIR value at the destination.

(4) $$r_{O1D,m}=d_{SD}{\left(\frac{\Gamma_{D}\lambda_{m,n}P_{O1,m}^{TX}}{P_{S}^{TX}}\right)}^{\frac{1}{\alpha }}.$$

**Figure 8:**
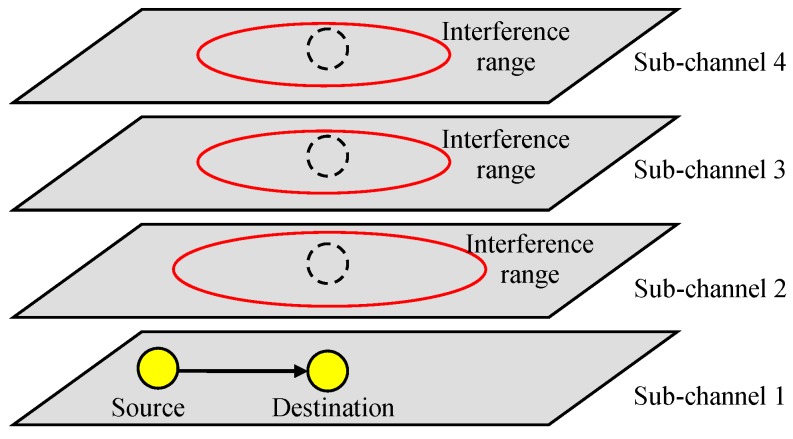
Other-channel interference.

Note that the interference range is not the same for all sub-channels and is dependent on $\lambda_{m,n}P_{O1,m}^{TX}$ for the $m^{th}$ sub-channel. As shown in Figure 8, the interference range for a sub-channel can be larger than others. 

If the $m^{th}$ channel interferer is located inside the circle of radius $r_{O1D,m}$ centered at the destination position $\left(x_{D},y_{D}\right)$, then the target SIR cannot be satisfied. Hence, the outage probability for the source–destination link resulting from the $m^{th}$-channel interferer can be approximately calculated as:

(5) $$\begin{array}{l} P_{SD,m}^{Outage}\approx \min\left\{1,\iint_{Interference_{SD}}f_{O,m}(x,y)dxdy\right\}\\ =\min\left\{1,\pi \;{r_{O1D,m}}^{2}{\tilde{f}}_{O,m}^{SD}(x_{D},y_{D})\right\}\\ =\min\left\{1,\pi \;{d_{SD}}^{2}{\left(\frac{\Gamma_{D}\lambda_{m,n}P_{O1,m}^{TX}}{P_{S}^{TX}}\right)}^{\frac{2}{\alpha }}{\tilde{f}}_{O,m}^{SD}(x_{D},y_{D})\right\} \end{array}$$

where $Interference_{SD}$ is the circular area of radius $r_{O1D,m}$ centered at the destination position $\left(x_{D},y_{D}\right)$, $f_{O,m}(x,y)$ is the interferer density at (x,y), and ${\tilde{f}}_{O,m}^{SD}(x_{D},y_{D})$ is the average interferer density of $Interference_{SD}$, defined as:

(6) $${\tilde{f}}_{O,m}^{SD}(x_{D},y_{D})\equiv \frac{1}{\pi \;r_{O1D,m}}\iint_{Interference_{SD}}f_{O,m}(x,y)dxdy.$$

Suppose that there is a hot-spot region for interferers with area $A_{Hotspot}$, and the interferer density of the hot-spot, denoted as $D_{O}^{Hotspot}$, is β times of that of other regions, then $D_{O}^{Hotspot}$ can satisfy

(7) $$D_{O}^{Hotspot}A_{Hotspot}+\frac{D_{O}^{Hotspot}}{\beta }(\pi \;R^{2}+A_{Hotspot})=1,$$

and, thus,

(8) $$D_{O}^{Hotspot}=\frac{\beta }{(\beta -1)A_{Hotspot}+\pi \;R^{2}}.$$

If interferers are distributed in a uniform distribution across the cell area, in other words, β=1 in Equation (8), then:

(9) $$f_{O,m}(x_{D},y_{D})=D_{O}^{Uniform}\equiv \frac{1}{\pi \;R^{2}},$$

and, thus, Equation (5) can be rewritten as follows:

(10) $$P_{SD,m}^{Outage}\approx \min\left(1,\frac{{r_{O1D,m}}^{2}}{R^{2}}\right)=\min\left\{1,\frac{{d_{SD}}^{2}}{R^{2}}{\left(\frac{\Gamma_{D}\lambda_{m,n}P_{O1,m}^{TX}}{P_{S}^{TX}}\right)}^{\frac{2}{\alpha }}\right\}.$$

Considering all M−1 sub-channels producing the other-channel interference to the $n^{th}$ sub-channel, the outage probability for the source–destination link with a uniform distribution of interferers can be expressed as follows:

(11) $$P_{SD}^{Outage}=1-\prod_{m=1,m\neq n}^{M}\left(1-P_{SD,m}^{Outage}\right)\approx 1-\prod_{m=1,m\neq n}^{M}\left(1-\min\left\{1,\frac{{d_{SD}}^{2}}{R^{2}}{\left(\frac{\Gamma_{D}\lambda_{m,n}P_{O1,m}^{TX}}{P_{S}^{TX}}\right)}^{\frac{2}{\alpha }}\right\}\right).$$

The outage probability can be reduced by repeated transmission through the relay. The signal power from the source at the relay is written as:

(12) $$P_{SR}^{RX}=K_{1}P_{S}^{TX}{d_{SR}}^{-\alpha },$$

and the interference power from the other-channel interferer 1,m at the relay can be expressed as:

(13) $$P_{O1R,m}^{RX}=K_{1}\lambda_{m,n}P_{O1,m}^{TX}{d_{O1R,m}}^{-\alpha }.$$

The SIR at the relay only considering the $m^{th}$-channel interferer, denoted as $SIR_{SR,m}$, can be expressed as follows:

(14) $$SIR_{SR,m}=\frac{P_{SR}^{RX}}{P_{O1R,m}^{RX}}=\frac{P_{S}^{TX}{d_{SR}}^{-\alpha }}{\lambda_{m,n}P_{O1,m}^{TX}{d_{O1R,m}}^{-\alpha }}.$$

The interference range, denoted as $r_{O1R,m}$, can be defined as the minimum $d_{O1R,m}$ satisfying $SIR_{SR,m}\geq \Gamma_{R}$, where $\Gamma_{R}$ is the required SIR at the receiver.

(15) $$r_{O1R,m}=d_{SR}{\left(\frac{\Gamma_{R}\lambda_{m,n}P_{O1,m}^{TX}}{P_{S}^{TX}}\right)}^{\frac{1}{\alpha }}.$$

The outage probability for the source–relay link from the $m^{th}$-channel interferer can be approximately calculated as follows:

(16) $$\begin{array}{l} P_{SR,m}^{Outage}\approx \min\left\{1,\iint_{Interference_{SR}}f_{O,m}(x,y)dxdy\right\}\\ \approx \min\left\{1,\pi \;{r_{O1R,m}}^{2}{\tilde{f}}_{O,m}^{SR}(x_{R},y_{R})\right\}\\ =\min\left\{1,\pi \;{d_{SR}}^{2}{\left(\frac{\Gamma_{R}\lambda_{m,n}P_{O1,m}^{TX}}{P_{S}^{TX}}\right)}^{\frac{2}{\alpha }}{\tilde{f}}_{O,m}^{SR}(x_{R},y_{R})\right\} \end{array}$$

where $Interference_{SR}$ is the circular area of radius $r_{O1R,m}$ centered at the relay position $\left(x_{R},y_{R}\right)$, and ${\tilde{f}}_{O,m}^{SR}(x_{R},y_{R})$ is the average interferer density of $Interference_{SR}$, defined as:

(17) $${\tilde{f}}_{O,m}^{SR}(x_{R},y_{R})\equiv \frac{1}{\pi \;r_{O1R,m}}\iint_{Interference_{SR}}f_{O,m}(x,y)dxdy.$$

If interferers are distributed in a uniform distribution over the cell area, in other words, Equation (9) holds, then Equation (16) can be rewritten as follows:

(18) $$P_{SR,m}^{Outage}\approx \min\left(1,\frac{{r_{O1R,m}}^{2}}{R^{2}}\right)=\min\left\{1,\frac{{d_{SR}}^{2}}{R^{2}}{\left(\frac{\Gamma_{R}\lambda_{m,n}P_{O1,m}^{TX}}{P_{S}^{TX}}\right)}^{\frac{2}{\alpha }}\right\}.$$

Hence, the outage probability for the source–relay link considering all M−1 sub-channels producing the interference to the $n^{th}$ sub-channel can be written as follows:

(19) $$P_{SR}^{Outage}=1-\prod_{m=1,m\neq n}^{M}\left(1-P_{SR,m}^{Outage}\right)\approx 1-\prod_{m=1,m\neq n}^{M}\left(1-\min\left\{1,\frac{{d_{SR}}^{2}}{R^{2}}{\left(\frac{\Gamma_{R}\lambda_{m,n}P_{O1,m}^{TX}}{P_{S}^{TX}}\right)}^{\frac{2}{\alpha }}\right\}\right).$$

Similarly, the interference range at the destination for the second transmission from the relay, expressed as *r*_*O*2*D*,*m*_, can be defined as:

(20) $$r_{O2D,m}=d_{RD}{\left(\frac{\Gamma_{D}\lambda_{m,n}P_{O2,m}^{TX}}{P_{R}^{TX}}\right)}^{\frac{1}{\alpha }}.$$

The outage probability for the relay-destination link from the other-channel interferer 2,m can be approximately calculated as follows:

(21) $$\begin{array}{l} P_{RD,m}^{Outage}\approx \min\left\{1,\iint_{Interference_{RD}}f_{O,m}(x,y)dxdy\right\}\\ =\min\left\{1,\pi \;{r_{O2D,m}}^{2}{\tilde{f}}_{O,m}^{RD}(x_{D},y_{D})\right\}\\ =\min\left\{1,\pi \;{d_{RD}}^{2}{\left(\frac{\Gamma_{D}\lambda_{m,n}P_{O2,m}^{TX}}{P_{R}^{TX}}\right)}^{\frac{2}{\alpha }}{\tilde{f}}_{O,m}^{RD}(x_{D},y_{D})\right\} \end{array}$$

where $Interference_{RD}$ is the circular area of radius $r_{O2D,m}$ centered at the destination position $\left(x_{D},y_{D}\right)$, and ${\tilde{f}}_{O,m}^{RD}(x_{D},y_{D})$ is the average interferer density of $Interference_{RD}$, defined as:

(22) $${\tilde{f}}_{O,m}^{RD}(x_{D},y_{D})\equiv \frac{1}{\pi \;r_{O2D,m}}\iint_{Interference_{RD}}f_{O,m}(x,y)dxdy.$$

If interferers are distributed in a uniform distribution over the cell area, then Equation (21) can be rewritten as follows:

(23) $$P_{RD,m}^{Outage}\approx \min\left(1,\frac{{r_{O2D,m}}^{2}}{R^{2}}\right)=\min\left\{1,\frac{{d_{RD}}^{2}}{R^{2}}{\left(\frac{\Gamma_{R}\lambda_{m,n}P_{O2,m}^{TX}}{P_{R}^{TX}}\right)}^{\frac{2}{\alpha }}\right\}.$$

Hence, the outage probability for relay-destination link can be written as follows:

(24) $$P_{RD}^{Outage}=1-\prod_{m=1,m\neq n}^{M}\left(1-P_{RD,m}^{Outage}\right)\approx 1-\prod_{m=1,m\neq n}^{M}\left(1-\min\left\{1,\frac{{d_{RD}}^{2}}{R^{2}}{\left(\frac{\Gamma_{D}\lambda_{m,n}P_{O2,m}^{TX}}{P_{R}^{TX}}\right)}^{\frac{2}{\alpha }}\right\}\right).$$

In this paper, we considered three different scenarios of D2D relaying, and the outage probability calculated using Equations (11), (19), and (24) according to the target scenario. Note that $P_{SD}^{Outage}$ in Equation (11) is a function of $d_{SD}$, $P_{SR}^{Outage}$ in Equation (19) is a function of $d_{SR}$, and $P_{RD}^{Outage}$ in Equation (24) is a function of $d_{RD}$. If other parameters are fixed, the outage probabilities can be calculated based on the source, relay, and destination positions. 

### 3.2. Scenario 1: Single Transmission though a Relay

In the first scenario, as shown in Figure 3, the source transmits data to the relay and the relay passes the data to the receivers in the group if the relay receives the data from the source successfully. Hence, the data reception at a receiver fails if the relay does not receive from the source successfully or the receiver does not receive from the relay successfully. With given source and relay positions $\left(x_{S},y_{S}\right)$ and $\left(x_{R},y_{R}\right)$, the outage probability for a receiver at location $\left(x_{D},y_{D}\right)$, denoted as $P_{Outage(1)}(x_{D},y_{D})$, can be written as:

(25) $$\begin{array}{l} P_{Outage(1)}(x_{D},y_{D})=1-\left\{\left(1-P_{SR}^{Outage}\right)\left(1-P_{RD}^{Outage}\right)\right\}\\ =P_{SR}^{Outage}+P_{RD}^{Outage}-P_{SR}^{Outage}P_{RD}^{Outage} \end{array}$$

where $P_{RD}^{Outage}$ is a function of $\left(x_{D},y_{D}\right)$. Hence, the outage probability for the group considering all receivers in the group can be expressed as:

(26) $$\begin{array}{l} P_{Outage(1)}^{Group}=\frac{1}{N_{D}}\iint_{Group}P_{Outage(1)}(x_{D},y_{D})f_{D}(x_{D},y_{D})dx_{D}dy_{D}\\ =\frac{1}{N_{D}}\iint_{Group}(P_{SR}^{Outage}+P_{RD}^{Outage}-P_{SR}^{Outage}P_{RD}^{Outage})f_{D}(x_{D},y_{D})dx_{D}dy_{D} \end{array}$$

where Group is the region of the group, $N_{D}$ is the number of destinations (receivers) in the group, and $f_{D}(x_{D},y_{D})$ is the destination density satisfying:

(27) $$N_{D}=\iint_{Gorup}f_{D}(x_{D},y_{D})dx_{D}dy_{D}.$$

If the destinations are uniformly distributed over Group, then the destination density $f_{D}(x_{D},y_{D})=D_{D}^{Uniform}$ satisfies:

(28) $$\iint_{Group}D_{D}^{Uniform}dx_{D}dy_{D}=N_{D},$$

and Equation (26) can be rewritten as:

(29) $$P_{Outage(1)}^{Group}=\frac{1}{A_{Group}}\iint_{Group}(P_{SR}^{Outage}+P_{RD}^{Outage}-P_{SR}^{Outage}P_{RD}^{Outage})f_{D}(x_{D},y_{D})dx_{D}dy_{D},$$

where $A_{Group}=\iint_{Group}1\;dx_{D}dy_{D}$.

### 3.3. Scenario 2: Repeated Transmission through a Relay

In the second scenario, as shown in Figure 4, the source transmits data to the receivers in the group, and the relay passes the data to the receivers if the transmission from the source to the relay is successful. With given source and relay positions $\left(x_{S},y_{S}\right)$ and $\left(x_{R},y_{R}\right)$, the outage probability for a receiver at location $\left(x_{D},y_{D}\right)$, denoted as $P_{Outage(2)}(x_{D},y_{D})$, can be represented as:

(30) $$\begin{array}{l} P_{Outage(2)}(x_{D},y_{D})=1-\left\{\left(1-P_{SD}^{Outage}\right)+P_{SD}^{Outage}\left(1-P_{SR}^{Outage}\right)\left(1-P_{RD}^{Outage}\right)\right\}\\ =P_{SD}^{Outage}P_{RD}^{Outage}+P_{SD}^{Outage}P_{SR}^{Outage}-P_{SD}^{Outage}P_{SR}^{Outage}P_{RD}^{Outage} \end{array}$$

where $P_{RD}^{Outage}$ and $P_{SD}^{Outage}$ are functions of $\left(x_{D},y_{D}\right)$. The outage probability considering all receivers in the group can be written as:

(31) $$\begin{array}{l} P_{Outage(2)}^{Group}=\frac{1}{N_{D}}\iint_{Group}P_{Outage(2)}(x_{D},y_{D})f_{D}(x_{D},y_{D})dx_{D}dy_{D}\\ =\frac{1}{N_{D}}\iint_{Group}(P_{SD}^{Outage}P_{RD}^{Outage}+P_{SD}^{Outage}P_{SR}^{Outage}-P_{SD}^{Outage}P_{SR}^{Outage}P_{RD}^{Outage})f_{D}(x_{D},y_{D})dx_{D}dy_{D}\\ =\frac{1}{A_{Group}}\iint_{Group}(P_{SD}^{Outage}P_{RD}^{Outage}+P_{SD}^{Outage}P_{SR}^{Outage}-P_{SD}^{Outage}P_{SR}^{Outage}P_{RD}^{Outage})dx_{D}dy_{D} \end{array}$$

assuming a uniform distribution of receivers.

### 3.4. Scenario 3: Repeated Transmission through a Successful Relay

In the third scenario, as shown in Figure 5, the source transmits data to the receivers in the group, and one of the candidate relays repeatedly transmits the data to the receivers, assuming that at least one of the candidate relays around $\left(x_{R},y_{R}\right)$ can receive data from the source. With given source and relay positions $\left(x_{S},y_{S}\right)$ and $\left(x_{R},y_{R}\right)$, the outage probability for a receiver at location $\left(x_{D},y_{D}\right)$, denoted as $P_{Outage(3)}(x_{D},y_{D})$, can be written as:

(32) $$\begin{array}{l} P_{Outage(3)}(x_{D},y_{D})=1-\left\{\left(1-P_{SD}^{Outage}\right)+P_{SD}^{Outage}\left(1-P_{RD}^{Outage}\right)\right\}\\ =P_{SD}^{Outage}P_{RD}^{Outage} \end{array}$$

since $P_{SR}^{Outage}=0$. The outage probability considering all receivers in the group can be expressed as:

(33) $$\begin{array}{l} P_{Outage(3)}^{Group}=\frac{1}{N_{D}}\iint_{Group}P_{Outage(3)}(x_{D},y_{D})f_{D}(x_{D},y_{D})dx_{D}dy_{D}\\ =\frac{1}{N_{D}}\iint_{Group}P_{SD}^{Outage}P_{RD}^{Outage}f_{D}(x_{D},y_{D})dx_{D}dy_{D}\\ =\frac{1}{A_{Group}}\iint_{Group}P_{SD}^{Outage}P_{RD}^{Outage}dx_{D}dy_{D} \end{array}$$

assuming a uniform distribution of receivers.

## 4. Simulation Results

In order to find the optimal relay position that minimizes the outage probability, we performed simulations according to the source and relay positions for the three relay scenarios shown in Figure 3, Figure 4 and Figure 5. We considered a circular cell with radius 100 m and assumed 25 sub-channels: one was the target sub-channel, and the other 24 sub-channels produced the other-channel interferences to the target sub-channel. In the target sub-channel, there was one D2D group with 30 receivers randomly distributed in a uniform distribution over a circular area of radius 30 m. For the other 24 sub-channels, there was one interferer per sub-channel at a time, and the other-channel interferers were randomly distributed in a uniform distribution over the cell area. The central position of the D2D group on the target sub-channel was (0 m, 0 m). The source was placed from (−30 m, 0 m) to (0 m, 0 m), and the relay was located from (−30 m, 0 m) to (30 m, 0 m) on the *x*-axis. The detailed simulation parameters are shown in Table 1.

**Figure 9:**
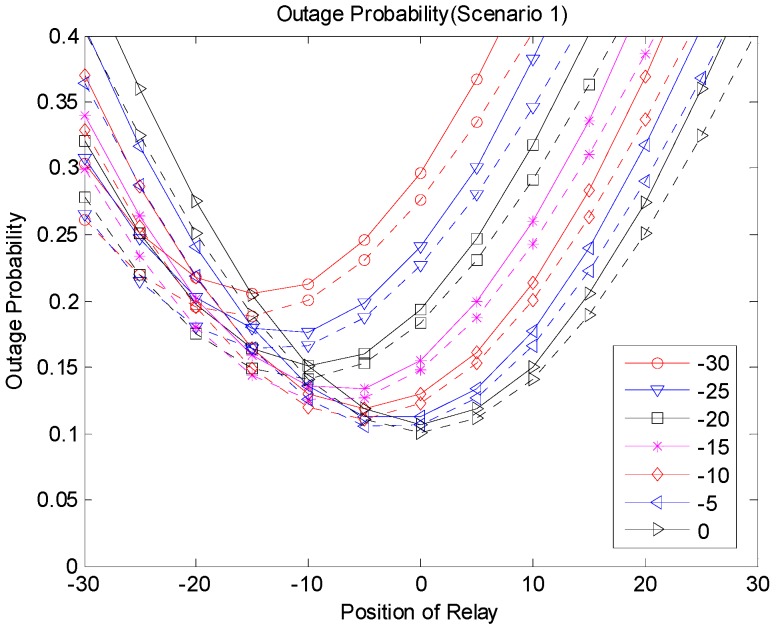
Outage probability (Scenario 1).

**Figure 10:**
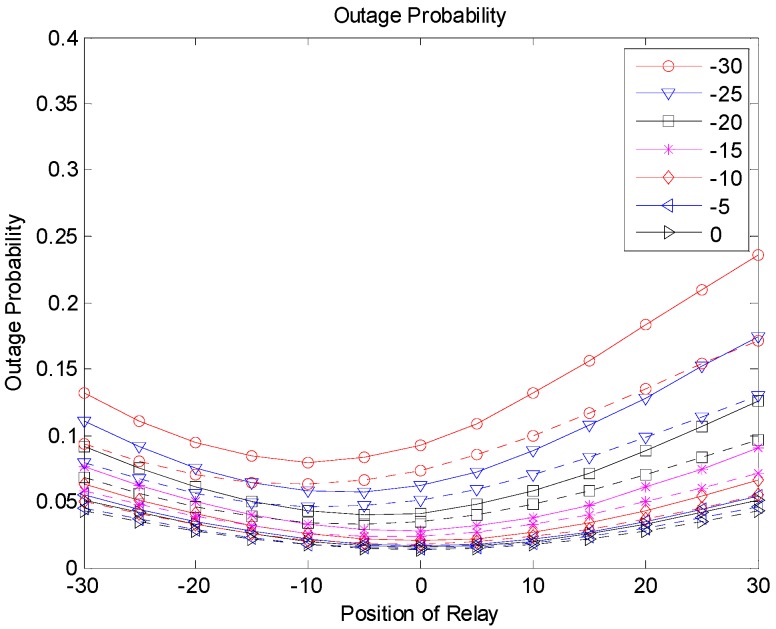
Outage probability (Scenario 2).

**Figure 11:**
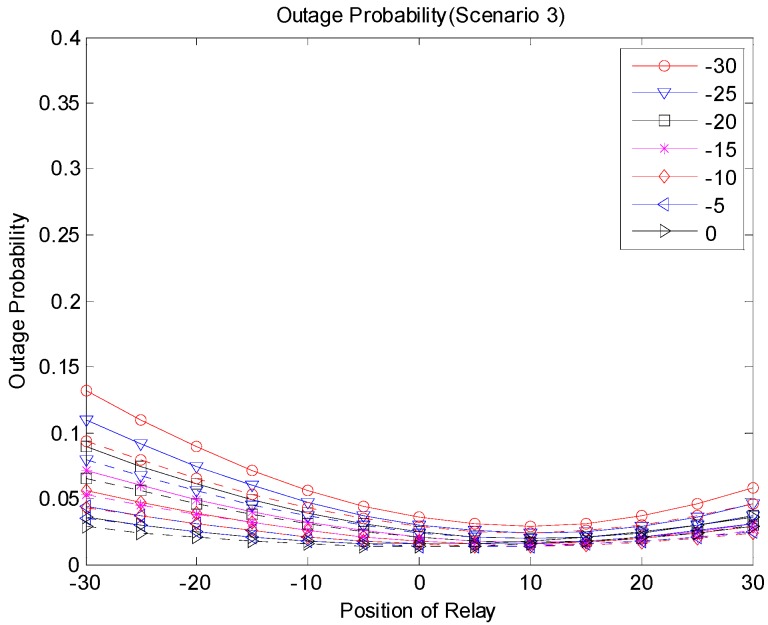
Outage probability (Scenario 3).

Figure 9, Figure 10 and Figure 11 show the outage probabilities with respect to the relay positions with different source locations for Scenarios 1, 2, and 3. The solid and dotted lines indicate the simulation and analytical results, respectively. For the analytical results, Equations (11), (19), (24), (29), (31), and (33) were used for calculating the outage probabilities. While only the interference from the nearest interferer was considered for the analytical results, the sum interference from the 24 other-channel interferers was used for the simulation results. Hence, the outage probabilities produced by the simulation were always greater than the corresponding outage probabilities calculated using the equations. There was no significant difference between the results by the analysis and by the simulation, and the equations were used to estimate the outage probability and to find the optimal relay position. The figures show that an appropriate relay position needs to be selected to reduce the outage probability. In addition, we could see that a better performance could be achieved by considering the direct transmission from the source as well as the transmission through the relay. The best result was generated with Scenario 3, where multiple candidates for the relay were considered, and one of the candidates that successfully received the packet performed the retransmission. If multiple relays transmit at the same time or consecutively, better results can be obtained, but the complexity may increase as well.

**Figure 12:**
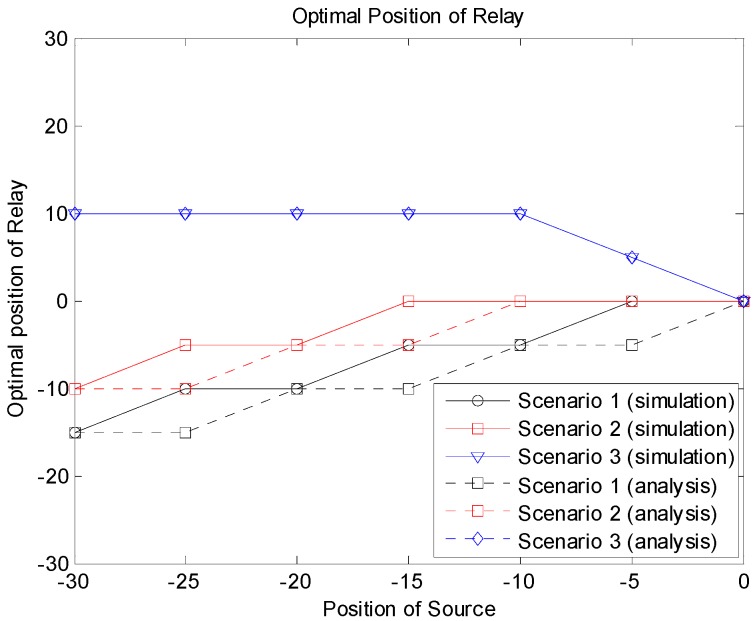
Optimal relay position.

Figure 12 shows the optimal relay positions producing the minimum outage probabilities according to the source locations for the three scenarios. From the simulation and analytical results, we can see that the optimal relay position was related to the location of the source as well as the system scenario. Note that the optimal position of the relay was not always the center of the group, especially when the source was near the edge of the group. In Scenario 1, the relay needed to be near the source, since the source–relay link should be reliable for the relay-based transmission. If direct transmission from the source to the destinations was allowed as in Scenario 2, the relay did not need to take care of receivers near the source, and the optimal position can be moved to the center of the group compared to Scenario 1. In Scenario 3, the relay may be placed far from the source, since we assumed that there were a sufficient number of candidates for relaying, and at least one of them could be successful. In this case, the relay can take care of receivers far from the source. 

**Figure 13:**
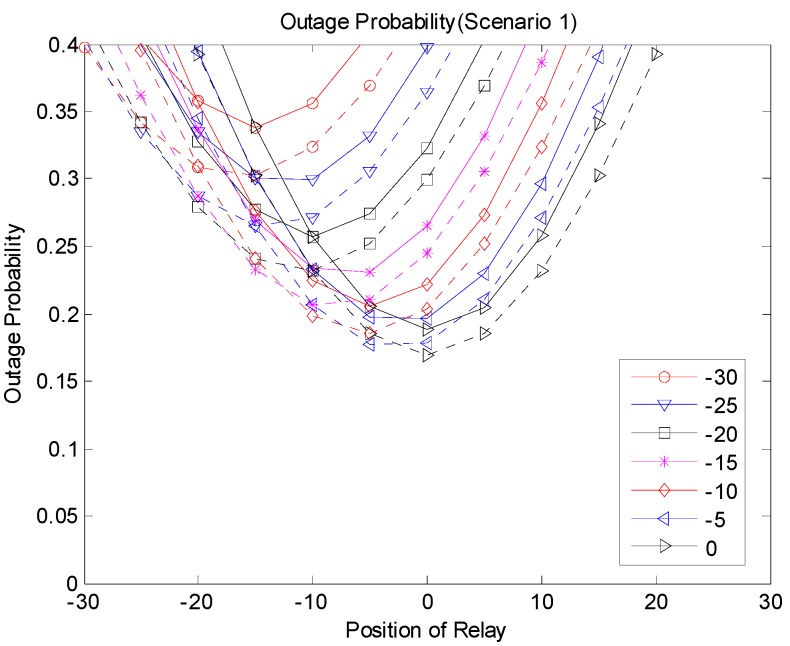
Outage probability (Scenario 1).

**Figure 14:**
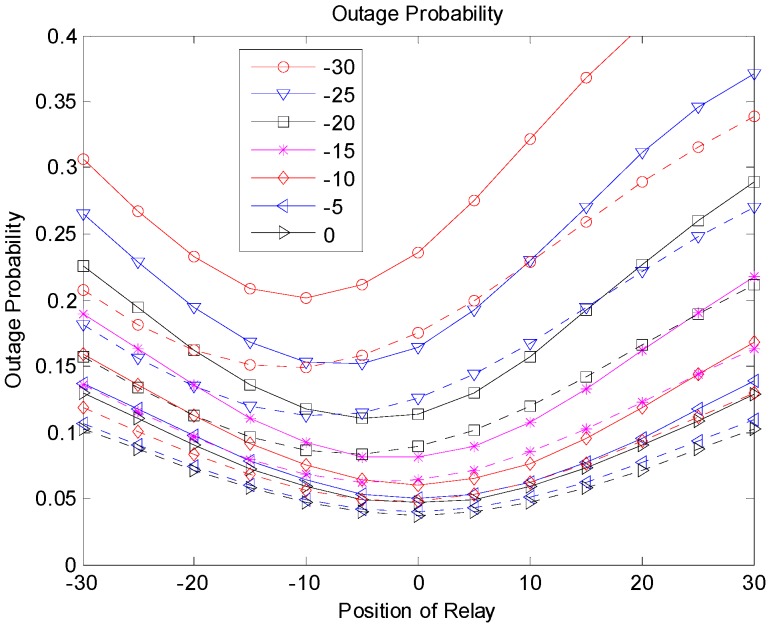
Outage probability (Scenario 2).

**Figure 15:**
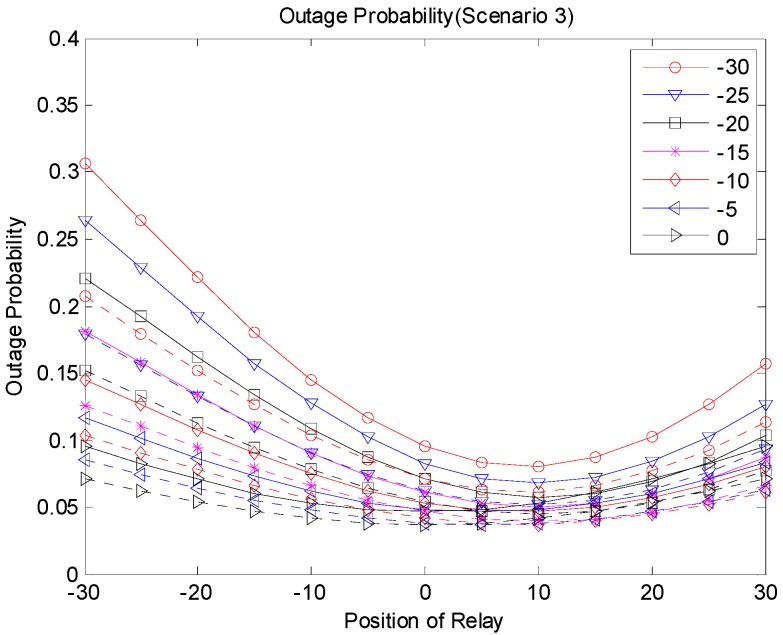
Outage probability (Scenario 3).

**Figure 16:**
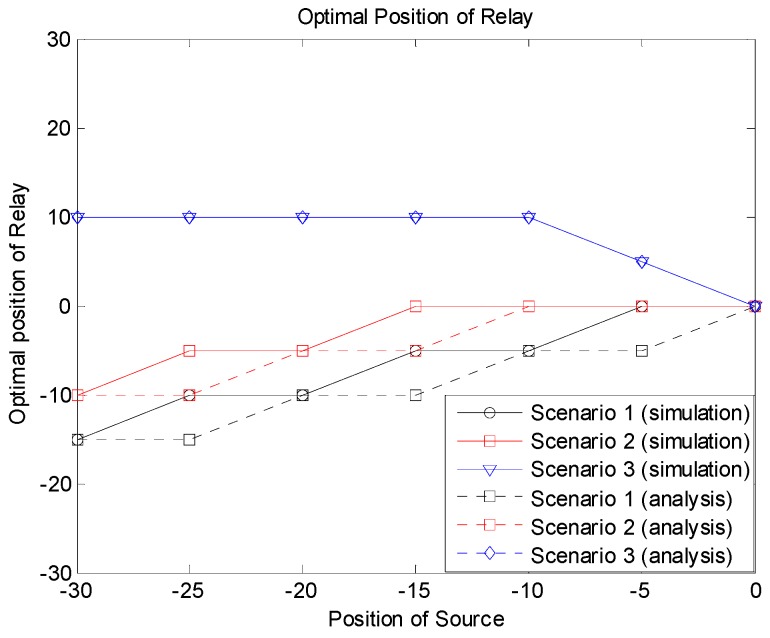
Optimal relay position.

The transmission power of devices may not be the same for all sub-channels and can be different depending on the sub-channels. This is also true for the interference effect between different sub-channels. Suppose that the target D2D group has a power limitation and the devices in the group use lower transmission power than devices in other sub-channels. We also considered the case in which the interference effect of some sub-channels was larger than that of other sub-channels. In this case, the influence of other-channel interference can become large. Figure 13, Figure 14 and Figure 15 show the simulation and analytical results using the simulation parameters in Table 2. Other simulation variables are the same as in Table 1. We can see that the outage probabilities were substantially increased because of the increased other-channel interference. However, the optimal relay position did not change significantly, and Figure 16 shows results similar to Figure 12. 

## 5. Conclusions

In OFDMA-based D2D systems, it is necessary to consider the interference from other sub-channels because of in-band emission. In this paper, we have discussed the performance degradation resulting from the interference from other sub-channels for OFDMA-based D2D group-casting systems. We considered three different scenarios of relaying, and performed analyses and simulations for the outage probabilities according to the relay positions. Although there is some approximation in the analysis, the difference between the results by the analysis and by the simulation is not significant, and the optimal relay location that minimizes the outage probability can be readily obtained using the analysis. 

The analytical and simulation results show that the optimal location of a relay is determined by the source position as well as the system scenario. Especially when the source is near or at the edge of the group, the optimal location of a relay in a group is not the center of the group. For some scenarios, the relay needs to be near the source for reliable transmission from the source to the relay. For other scenarios, the relay can be placed away from the source to take care of receivers far from the source. 

In this paper, we focused on the outage resulting from the interference from other sub-channels, and other performance-degradation factors were not considered, including collisions between intra-group devices, insufficient signal power from fading, and co-channel interference from other devices on the same sub-channel. More rigorous theoretical analyses and accurate simulations need to be performed with more practical assumptions. In the future, we will also consider multiple relays for retransmission to further improve the performance. 

## Figures and Tables

**Table 1:** Simulation parameters for Figure 9, Figure 10, Figure 11 and Figure 12.

Parameters	Values
Cell radius (R)	100 m
Group radius	30 m
Number of sub-channels	25
Number of interferers per sub-channel	1
Number of receivers in D2D group	30
Target signal-to-interference ratio (SIR) at relay ($\Gamma_{R}$)	10 dB
Target SIR at destination ($\Gamma_{D}$)	10 dB
Interference effect to other sub-channels ($\lambda_{m,n}$, m≠n)	−30 dB
Source transmission power ($P_{S}^{TX}$)	20 dBm
Relay transmission power ($P_{R}^{TX}$)	20 dBm
Interferer transmission power ($P_{O1,m}^{TX},P_{O2,m}^{TX}$)	20 dBm
Path loss exponent between two devices (α)	4
Center position of D2D group	(0 m, 0 m)
Source position ($x_{S},y_{S}$)	(30~0 m, 0 m)
Relay position ($x_{R},y_{R}$)	(−30~30 m, 0 m)

**Table 2:** Simulation parameters for Figure 13, Figure 14, Figure 15 and Figure 16.

Parameters	Values
Interference effect to other sub-channels ($\lambda_{m,n}$, m≠n)	−24 dB for 6 sub-channels −30 dB for 18 sub-channels
Source transmission power ($P_{S}^{TX}$)	17 dBm
Relay transmission power ($P_{R}^{TX}$)	17 dBm
Interferer transmission power $P_{O1,m}^{TX},P_{O2,m}^{TX}$)	20 dBm
